# Comparative analysis of vascular closure devices for neuroendovascular procedures using Perclose versus Angioseal

**DOI:** 10.1016/j.heliyon.2024.e39975

**Published:** 2024-10-30

**Authors:** Takehiro Uno, Kouichi Misaki, Taishi Tsutsui, Tomoya Kamide, Mitsutoshi Nakada

**Affiliations:** Department of Neurosurgery, Kanazawa University School of Medicine, Ishikawa, Japan

**Keywords:** Neuroendovascular treatment, Perclose ProGlide, Angioseal, Vascular closure device, Puncture-site complication

## Abstract

**Background:**

The common femoral artery is most frequently accessed for neuroendovascular procedures. Occasionally, postoperative hemostasis is difficult to attain. We comparatively analyzed the vascular closure devices, Angioseal and Perclose, and evaluated the puncture-site complications.

**Methods:**

357 patients who underwent endovascular treatment via femoral artery puncture and achieved hemostasis using a device were included. We studied the hemostatic method and associated puncture-site complications for various conditions. Ordinal logistic multiple regression analysis was performed for age, sex, sheath diameter, hemostatic method, number of antiplatelet agents, and object disease to identify the factors associated with puncture-site complications.

**Results:**

Angioseal was used in 233 cases, and Perclose in 124 cases. Non-surgical complications were observed in 7.3 % and 0.8 % patients respectively. Complications requiring surgery occurred in 0.9 % of Angioseal, while none of Perclose. The complication rate at the puncture site was 10 % for unruptured aneurysms, 2.3 % for ruptured aneurysms, 3.5 % for carotid artery stenosis, and 4.0 % for acute arterial occlusive disease. Complication-related factors (*P* < 0.05) were fewer for Perclose (*P* = 0.0013,OR = 0.09) and higher for unruptured aneurysms (*P* = 0.0085, OR = 3.62).

**Conclusions:**

Perclose is a vascular closure device with rare puncture-site complications for neurosurgical diseases. Unruptured aneurysm cases require careful attention to puncture-site complications.

## Highlights

• Angioseal was used in 233 cases and Perclose in 124, with non-surgical complications observed in 7.3% and 0.8% patients, respectively.
• Perclose is a vascular closure device with rare puncture-site complications for neurosurgical diseases.
• Unruptured aneurysm cases require careful attention to puncture-site complications.

## Abbreviations

CFA: Common femoral artery
DAVF: Dural arteriovenous fistula
MC: manual compression
VCD: vascular closure devices

## 1 Introduction

The common femoral artery (CFA) is a commonly accessed site for neuroendovascular procedures owing to its large diameter, superficial nature, and fixation by surrounding tissue [1]. The risk of long-term bleeding after catheter or sheath removal is reported to be low because of the ease of manual compression(MC) [1]. However, occasionally, there are cases in which postoperative hemostasis is challenging to achieve. Vascular access site complications such as local hemorrhage, hematoma, pseudoaneurysm, arteriovenous fistula, and retroperitoneal hematoma reportedly account for 4.2 % cases [2], making safe and efficient closure of vascular access sites crucial to avert the compromising ADL after endovascular treatment. Vascular closure devices (VCD), with a lower rate of serious complications and higher success rate, have been reported to improve the time required for hemostasis and ambulation compared to MC, with comparable complication rates [3], thereby contributing to patient comfort and satisfaction after percutaneous vascular procedures. Commonly used VCDs are either collagen-based devices, such as Angioseal or suture-mediated devices, such as Perclose ProGlide. Approximately 3.5 %–16 % of puncture-site complications have been reported with Angioseal, and further improvement in outcomes is desired [4,5]. Recently, the use of Perclose ProGlide (Abbot Vascular, USA) in neuroendovascular treatment has increased. Perclose is a VCD that deploys percutaneous sutures to close the CFA incision site. Although the usage of Perclose has been reviewed in the field of cardiovascular diseases, it is seldomly reported in the field of neuroendovascular diseases. Even among endovascular treatments, the devices used for cardiovascular and neurosurgical diseases differ. In addition, factors such as patient characteristics and perioperative protocols for antithrombotic therapy also vary significantly, highlighting the importance of analyzing these cases separately. In this study, we compared the safety and efficacy of Perclose with those of Angioseal. Furthermore, we also examined the risk factors related to puncture-site complications, taking patient information into account.

## 2 Material and methods

### 2.1 Patient data

Of the patients who underwent endovascular treatment by CFA puncture between January 2019 and September 2022 at our hospital, 357 cases of hemostasis attained using a VCD were included. We retrospectively analyzed puncture site complications in patients following endovascular treatment using medical record data. Incidences of puncture-site complications and hemostatic method were assessed comparatively for various conditions. Puncture-site complications were defined as cases in which bleeding or ischemia occurred after attaining hemostasis and required additional treatment. Minor complications were defined as those in which hemostasis was achieved with MC and major complications were those in which surgical treatment was indicated. To identify the factors associated with puncture-site complications, ordinal logistic multiple regression analysis was performed for age, sex, sheath size, number of antiplatelet medications taken, hemostatic method, and object disease. Written informed consent was obtained from all patients. This study was performed according to the guidelines of the Internal Review Board of Kanazawa University and was approved by the Medical Ethics committee of Kanazawa University (approval numbers 114168-1; approved December 21, 2022).

### 2.2 Statistical analysis

Continuous data and the number of observations are reported as the mean ± standard deviation for continuous variables (frequency, %). We used ordinal logistic multiple regression analysis with a stepwise method for the multivariate analysis. Statistical significance was set at *P* < 0.05. Statistical analyses were performed using SPSS software (IBM SPSS Statistics 24, Chicago, IL, USA).

## 3 Results

The mean age of our patients was 68.1 years. Of these, 173 were male and 184 were female. The underlying conditions for treatment included unruptured aneurysms in 140 patients, ruptured aneurysms in 44 patients, carotid artery stenosis in 57 patients, acute arterial occlusion in 72 patients, and dural arteriovenous fistulas (DAVF) in 44 patients (Table 1).

**Table 1:** Patient characteristics.

	n（%）or mean ± SD
Age （yr）	68.1 ± 12.9
Male	173 (48.5)
Elderly (>70yr)	194 (54.3)
Large sheath (>6Fr)	171 (47.9)
1 antiplatelet agent	2 (0.6)
2 antiplatelet agent	237 (66.4)
Perclose	233 (65.3)
Angioseal	124 (34.7)
Unruptured aneurysm	140 (39.2)
Ruptured aneurysm	44 (12.3)
Carotid artery stenosis	57 (16.0)
Acute artery occlusion	72 (20.2)
DAVF	44 (12.3)

DAVF; Dural arteriovenous fistula.

Major complications requiring surgical treatment were observed in 2 (0.9 %) in the Angioseal group and none in the Perclose group; furthermore, minor complications were observed in 17 (7.3 %) and 1 (0.8 %) case of the Angioseal and Perclose group, respectively. Minor complications included unruptured aneurysms in 13 (9.3 %), ruptured aneurysms in one (2.3 %), carotid artery stenosis in 2 (3.5 %), and acute arterial occlusion in 2 (2.7 %) patients, and no cases of DAVF (Table 2). Major complications included unruptured aneurysm in one (0.7 %), acute arterial occlusion in one (1.4 %), and no ruptured aneurysm, carotid artery stenosis, brain tumor, or DAVF (Table 3). The patient with an unruptured cerebral aneurysm was a 70-year-old woman who was diagnosed with CFA occlusion 6 h after surgery when a cold sensation appeared in her lower extremities due to CFA occlusion. The patient with acute arterial occlusion was an 80-year-old man who developed a pseudoaneurysm in his right groin, 9 days after the treatment and was treated surgically by a vascular surgeon the following day. Multivariate analysis of complication-related factors using a stepwise method (*P* < 0.15) was lower in Perclose (*P* = 0.025, OR = 0.10) and higher in unruptured aneurysms (*P* = 0.013, OR = 3.55) (Table 4). Statistical analysis showed fewer complications in cases of Perclose use.

**Table 2:** Minor complication.

	Sheath size (Fr)	Unruptured aneurysm			Ruptured aneurysm			Carotid artery stenosis			Acute arterial occlusion			DAVF			Total		
		C	N	%	C	N	%	C	N	%	C	N	%	C	N	%	C	N	%
Angioseal	6.6 ± 0.9	12	94	12.8	1	33	3.0	2	37	5.4	2	46	4.3	0	23	0	17	233	7.3
Perclose	7.6 ± 0.8	1	46	2.2	0	11	0	0	20	0	0	26	0	0	21	0	1	124	0.8
Total		13	140	9.3	1	44	2.3	2	57	3.5	2	72	2.7	0	44	0	18	357	5.0

C; Complication, DAVF; Dural arteriovenous fistula, N; Number. Values are shown as mean ± SD when appropriate.

**Table 3:** Major complication.

	Sheath size (Fr)	Unruptured aneurysm			Ruptured aneurysm			Carotid artery stenosis			Acute arterial occlusion			DAVF			Total		
		C	N	%	C	N	%	C	N	%	C	N	%	C	N	%	C	N	%
Angioseal	6.6 ± 0.9	1	94	1.1	0	33	0	0	37	0	1	46	2.2	0	23	0	2	233	0.9
Perclose	7.6 ± 0.8	0	46	0	0	11	0	0	20	0	0	26	0	0	21	0	0	124	0
Total		1	140	0.7	0	44	0	0	57	0	1	72	1.4	0	44	0	2	357	0.6

C; Complication, DAVF; Dural arteriovenous fistula, N; Number. Values are shown as mean ± SD when appropriate.

**Table 4:** Risk factors associated with puncture site complications.

	Multivariate analysis		
	OR	95 % CI	P value
Male		Not evaluated	–
Elderly (>70yr)		Not evaluated	–
Large sheath (>6Fr)		Not evaluated	–
One antiplatelet agent		Not evaluated	–
Two antiplatelet agent		Not evaluated	–
Unruptured aneurysm	3.55	1.30–9.65	0.013
Ruptured aneurysm		Not evaluated	–
Carotid artery stenosis		Not evaluated	–
Acute artery occlusion		Not evaluated	–
Perclose	0.10	0.01–0.75	0.025

## 4 Discussion

**Fig. 1:**
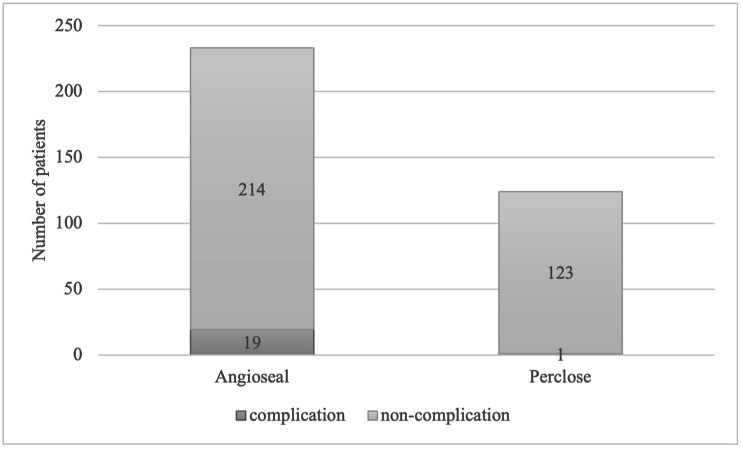
The stacked bar graph shows the number of patients with puncture site complications per vascular closure device. The complication rates for Angioseal and Perclose were 8.2 % and 0.8 %, respectively, with Angioseal having a higher rate.

**Fig. 2:**
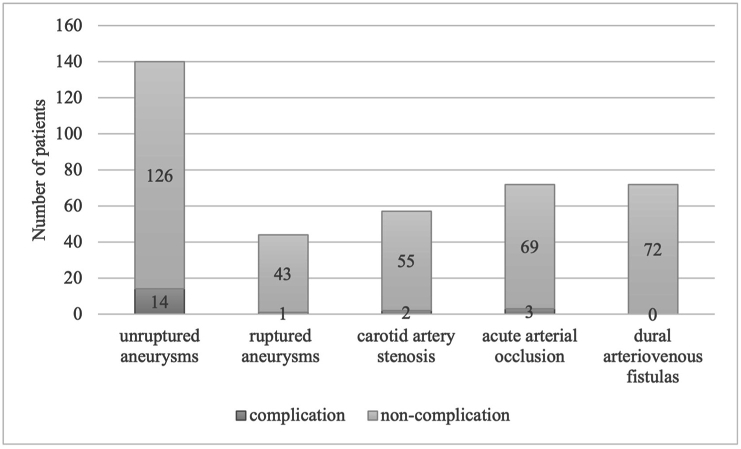
Stacked bars indicate the number of patients with puncture site complications per disease. The complication rates for each condition were 10 % for unruptured aneurysms, 2.3 % for ruptured aneurysms, 3.5 % for carotid artery stenosis, 4.2 % for acute arterial occlusion, and 0 % for dural arteriovenous fistulas, with unruptured aneurysms showing the highest rate.

This study compared the vascular closure devices, Angioseal and Perclose, focusing on puncture site complications. Angioseal was used in 233 cases, while Perclose was used in 124 cases. Surgical complications occurred in 8.2 % of the Angioseal group compared to 0.8 % in the Perclose group (Fig. 1). The highest complication rate at the puncture site was 10 % for unruptured aneurysms (Fig. 2).

In this study, the minor complication rates were 2.3 % for ruptured aneurysms and 9.3 % for unruptured aneurysms. At our institution, the patients are initiated on two antiplatelet agents, 2 weeks before the scheduled surgery for unruptured aneurysms and carotid artery stenosis. Previously, antithrombotic medication was regarded as a risk factor for puncture-site complications [6]. Although the difference was not statistically significant in this study, it may still be relevant. In contrast, patients with carotid artery stenosis receiving antiplatelet agents had a major complication rate of 1.4 %, which was comparable to that of ruptured aneurysms. All patients with carotid artery stenosis were evaluated preoperatively to examine the access routes to exclude patients with shaggy aortas and prevent other complications. In patients with severe atherosclerosis, carotid artery stenting via the brachial approach or carotid endarterectomy was performed, which may be related to the influence of case selection. Patients with ruptured aneurysms were advised complete postoperative bedrest. The difference in the degree of postoperative rest and the hypercoagulable state in patients with subarachnoid hemorrhage [7] may contribute in reducing puncture-site bleeding. In addition, in patients with acute arterial occlusion, 1.4 % of them had major complications and 2.7 % suffered minor complications. The treatment is urgent and time-sensitive, and lack of preparation may add to puncture-site complications.

Of the cases reviewed in this study, nine (7.3 %) of the Perclose devices failed during the period of hemostasis. Failures are cases that required manual compression immediately after hemostasis or a second device in case of failure with a wire still in place. Five cases of knot failure, three cases of thread loss due to poor needle insertion, and one case of disconnection during traction in the final stage were among the failures. Perclose device failures were reported to be 5–6% when used in "preclose" method [8,9]. The causes of device failure in previous reports included arterial division at the site of device entry, failure of needle puncture due to calcification of the vessel, and failure to form or deliver a knot [[10], [11], [12], [13], [14]]. Dissection of the subcutaneous tissue along the sheath at the puncture site was considered critical. Cases in which the knot could not be delivered were those in which the dissection was insufficient, and the presence of connective tissue made the knot challenging to tie. Insufficient dissection may also be associated with obesity, which has been reported as a risk factor for puncture-site bleeding [15]. To reduce complications at the puncture site, the subcutaneous tissue around the sheath should be appropriately dissected circumferentially along the puncture site of the artery. In addition, resistance of the vessels at the time of puncture was observed in all cases of poor needle insertion possibly due to calcification. Calcification of the CFA is also considered a risk factor for puncture-site complications [9,11]. Other hemostatic methods should be considered in cases where resistance is felt at the time of arterial puncture or in cases in which calcification of the CFA is observed on computed tomography or echocardiography before surgery.

Previously, large sheath diameter has been reported to be a risk factor for puncture-site complications in Perclose cases [9,[16], [17], [18], [19], [20]]. In our study, the sheath diameter was not significantly associated with puncture-site complications. Previous studies were mostly conducted in the cardiovascular field and included sheath sizes of 20Fr or larger. In the neuroendovascular field, the maximum recommended sheath size is approximately 9Fr, and all studies at our institution have been conducted using sheaths of 8Fr or8 smaller. The sheath diameter may not be related to puncture-site complications if the device size is0 08the same as that used in the neuroendovascular field. In close proximity cases, studies reported tha18t major complications ranged from 0.3 % to 2.5 % and minor complications from 0.6 % to 2.5 % [9,15], but in our study, major complications were 0 % and minor complications were as low as 0.8 %. A low rate of puncture-site complications in the neuroendovascular field could be owing to the sheath size used [15] or distinct disease characteristics.

### 4.1 Limitations

Our study had a small sample size, and the cases were from a single institution which further limited the number of target conditions. We began using Perclose in 2021, which is currently the first choice for treatment, prior to which Angioseal was used. The cases were not randomized here adding to the confounding factors and retrospective study that does not follow standardized protocols. In this study, hemostasis was performed by four neurosurgeons specialized in cerebral angiography and endovascular treatment. However, Perclose is a complicated procedure that requires familiarity with the technique, and the results may have been affected by the shortfall in treating a number of cases with Perclose, the years of practice, and the degree of proficiency in endovascular treatment. Therefore, operators experience of VCD may impact outcomes.

## 5 Conclusions

Perclose is a useful VCD for treating neuroendovascular diseases. In addition, unruptured aneurysms require careful attention to avoid puncture-site complications.

## CRediT authorship contribution statement

**Takehiro Uno:** Writing – original draft, Investigation, Funding acquisition, Data curation. **Kouichi Misaki:** Writing – review & editing, Validation, Conceptualization. **Taishi Tsutsui:** Writing – review & editing, Data curation. **Tomoya Kamide:** Writing – review & editing, Data curation. **Mitsutoshi Nakada:** Writing – review & editing, Supervision.

## Ethics

All procedures performed in studies involving human participants were in accordance with the ethical standards of the institutional and/or national research committee and with the 1964 Helsinki Declaration and its later amendments or comparable ethical standards.

## Data availability statement

The data that support the findings of this study are available from the corresponding author, KM, upon reasonable request.

## Funding

This work was supported by JSPS KAKENHI Grant Number JP23K15660.

## Declaration of competing interest

The authors declare that they have no conflict of interest.
